# Nanostring-Based Identification of the Gene Expression Profile in Trigger Finger Samples

**DOI:** 10.3390/healthcare9111592

**Published:** 2021-11-20

**Authors:** Ravindra Kolhe, Umar Ghilzai, Ashis K. Mondal, Chetan Pundkar, Pankaj Ahluwalia, Nikhil S. Sahajpal, Jie Chen, Carlos M. Isales, Mark Fulcher, Sadanand Fulzele

**Affiliations:** 1 Department of Pathology, Augusta University, Augusta, GA 30912, USA; rkolhe@augusta.edu (R.K.); amondal@augusta.edu (A.K.M.); pahluwalia@augusta.edu (P.A.); nsahajpal@augusta.edu (N.S.S.); 2 Department of Orthopedics, Augusta University, Augusta, GA 30912, USA; UGHILZAI@augusta.edu (U.G.); MAFULCHER@augusta.edu (M.F.); 3 Department of Pathology, Auburn University, Auburn, AL 36849, USA; cyp0003@auburn.edu; 4 Division of Biostatistics & Data Sciences, DPHS, Augusta University, Augusta, GA 30912, USA; jiechen@augusta.edu; 5 Center for Healthy Aging, Augusta University, Augusta, GA 30912, USA; CISALES@augusta.edu; 6 Division of Endocrinology, Diabetes and Metabolism, Augusta University, Augusta, GA 30912, USA; 7 Cellular Biology and Anatomy, Augusta University, Augusta, GA 30912, USA

**Keywords:** trigger finger, pain, gene expression, Nanostring

## Abstract

Trigger finger is a common yet vastly understudied fibroproliferative hand pathology, severely affecting patients’ quality of life. Consistent trauma due to inadequate positioning within the afflicted finger’s tendon/pulley system leads to cellular dysregulation and eventual fibrosis. While the genetic characteristics of the fibrotic tissue in the trigger finger have been studied, the pathways that govern the initiation and propagation of fibrosis are still unknown. The complete gene expression profile of the trigger finger has never been explored. Our study has used the Nanostring nCounter gene expression assay to investigate the molecular signaling involved in trigger finger pathogenesis. We collected samples from patients undergoing trigger finger (*n* = 4) release surgery and compared the gene expression to carpal tunnel tissue (*n* = 4). Nanostring nCounter analysis identified 165 genes that were differentially regulated; 145 of these genes were upregulated, whereas 20 genes were downregulated. We found that several collagen genes were significantly upregulated, and a regulatory matrix metalloproteinase (MMP), MMP-3, was downregulated. Bioinformatic analysis revealed that several known signaling pathways were dysregulated, such as the TGF-β1 and Wnt signaling pathways. We also found several novel signaling pathways (e.g., PI3K, MAPK, JAK-STAT, and Notch) differentially regulated in trigger finger. The outcome of our study helps in understanding the molecular signaling pathway involved in the pathogenesis of the trigger finger.

## 1. Introduction

Trigger finger, also known as stenosing tenosynovitis, is a musculoskeletal disorder in which a finger gets “locked” in either a flexed or extended position due to the disproportion between the diameter of that finger’s flexor tendon and pulley system. Friction is generated as the flexor tendon glides through the pulley and creates an intratendinous lump, leading to common manifestations of the trigger finger [1,2]. Histologically, the normal musculoskeletal connective tissue found in the pulley system shows abnormal characteristics with small collagen fibers and abundant extracellular matrix (ECM) proteins, along with fibrocartilage metaplasia [3,4,5]. The most common symptom of this disorder is the “catching” of the finger in question in a flexed position, in addition to pain, clicking, and loss of motion in the finger. These symptoms characterize trigger finger as one of the most common causes of hand pain in adults. Although not defined as a life-threatening condition, the pain and discomfort due to untreated trigger fingers are reported to cause significant debilitation for patients [6,7]. Treatment options of those suffering from trigger finger vary between noninvasive and invasive options, depending upon the severity of the condition. Patients can opt for treatment that ranges from noninvasive splinting, corticosteroid injections, shockwave therapy, or invasive surgical release [8,9]. Studies have suggested that the best and most cost-effective treatment of trigger finger is an immediate surgical release or corticosteroid injections followed by an eventual surgical release [9].

The gene expression profiling of the trigger finger pathogenesis has not been fully explored and only investigated a few selected extracellular matrix (ECM)-related genes. Previously, our group [10] and others [11] have reported elevated levels of ECM (collagen type 1a1, collagen type 3a1, aggrecan, and biglycan) and downregulation of MMP-3 and TIMP-3 [12]. The changes in expression levels of these genes can result in ECM imbalance and possibly eventual molecular pathogenesis of the trigger finger. The studies mentioned above [10,11,12] focused on ECM and growth factors genes, which did not provide a complete gene expression profile of trigger finger pathogenesis. Our study attempted to investigate the comprehensive gene expression profile of ECM and inflammatory signaling pathways using Nanostring technology to uncover possible trigger finger molecular etiologies.

The Nanostring nCounter Gene Expression Assay is a high-fidelity, simple protocol that allows the detection of up to 800 genes in a single reaction. The assay digitally detects mRNA molecules of interest using specific probes. The first probe anneals to the 5′-end of the target gene, which enables molecular barcoding downstream. The second probe carries a biotin marker which allows the anchoring of the gene for downstream detection. The genes are then immobilized and analyzed using their corresponding color codes to identify the expression levels of each of the molecules of interest [13]. The Nanostring nCounter Gene Expression Assay removed the need for any tedious enzymatic reactions and has also been proven simpler and more effective compared to other alternatives such as SYBR Green real-time PCR [14,15,16,17,18,19,20]. The Nanostring nCounter Gene Expression Assay tool has also been previously used to profile pathogenic gene expression profiles during infection [21,22]. We aimed to understand the molecular pathways that lead to fibrotic tissue generation in trigger finger. To the best of our knowledge, no studies have investigated the full breadth of differential gene expression in the trigger finger condition.

In this study, we collected tissue samples from the patients visited for trigger finger and carpal tunnel release surgery. We considered the carpal tunnel tissue samples as a control. Total RNA was isolated, and the Nanostring nCounter Gene Expression Assay was performed. We identified several differentially regulated genes in the trigger finger. Our goal for this study was to identify possible molecular pathways that lead to the pathogenesis of the trigger finger. Identifying potential genes or biomarkers would serve as valuable information for the future treatment of patients suffering from trigger finger.

## 2. Materials and Methods

### 2.1. Ethical Approval and Informed Consent

All relevant national policies and institutional regulations were followed according to the Helsinki Declaration to conduct our research on human tissue samples. All steps of this protocol were reviewed, audited, and accepted by the Augusta University Institutional Review Board (IRBNet ID: 611626-4) or the equivalent governing body. Informed consent was obtained from all patients undergoing the indicated procedures.

### 2.2. Obtaining Patient Samples

Experimental tissue specimens were collected from the patients undergoing A1 pulley trigger finger release surgery for symptomatic trigger finger (TF) at the Augusta University Medical Center. Control tissue specimens were collected from the patients undergoing carpal tunnel release surgery at the Augusta University Medical Center. We confirmed that patients with carpal tunnel syndrome had no clinical evidence or history of previous trigger finger before collecting tissue samples. Patient characteristics are described in Table 1. All surgeries were performed by a practicing, board-certified hand and upper-extremity surgeon employed by the Department of Orthopedic Surgery. Patient samples were then classified into two groups: trigger finger (*n* = 4) and carpal tunnel syndrome (*n* = 4) as the control samples. Specimens were then directly transported from the operating room to the laboratory. They were all snap-frozen and kept at −80 °C [10].

### 2.3. RNA Isolation and NanoString’s nCounter XT Gene Expression Assay

Total RNA was isolated from tissues as per the published method [10]. In brief, the frozen tissue samples were ground with liquid N_2_ using a mortar and pestle. The RNA was isolated using Trizol as per the manufacturer’s instructions. The quality of the RNA was measured by absorbance at 260 nm and 280 nm (Helios-Gamma, Thermo Spectronic, Rochester, NY, USA). We used NanoString’s nCounter (NanoString Technologies, Inc. 530 Fairview Ave N, Seattle, WA, USA) technology for gene expression comparison between different groups at GEM labs, LLC, (Department of Pathology, Augusta University). NanoString’s nCounter technology is based on digital detection and direct molecular barcoding of individual target molecules through the use of a unique probe pair for each target of interest. The probe pair consists of a color-coded Reporter probe, which carries the visible signal on its 5′ end, and a Capture probe, which carries a biotin moiety on the 3′ end. One hundred nanograms of total RNA (OD260/280 ratio 1.7–2.2) is hybridized overnight (>12 h) with reporter and capture code set at 65 °C, and excess probes are washed away using a two-step magnetic bead-based purification on an nCounter instrument. Finally, the purified target-probe complexes are eluted off the beads, immobilized on the cartridge, and aligned for data collection. Data collection was performed using epifluorescence microscopy and CCD capture technology on an nCounter instrument to yield hundreds of thousands of target molecule counts. Digital images are processed within the nCounter instrument, and the Reporter Probe counts are tabulated in a comma separated value (CSV) format for convenient data analysis with NanoString’s free nSolver™ Analysis Software V.3 (NanoString Technologies, Inc. 530 Fairview Ave N, Seattle, WA, USA).

### 2.4. Statistical Method

In this study, the nCounter PanCancer Pathways panel that included 770 genes from 13 canonical pathways (see Supplementary Table S1 for gene and probe information). These gene sets covered diverse biological pathways such as Notch, Wnt, Hedgehog, chromatin modification, transcriptional misregulation, DNA damage repair, TGFβ, MAPK, JAK-STAT, PI3K, Ras, cell cycle, and apoptosis. The samples were read at 555 FOV (Field of view) and resulting RCC data files were analyzed for QC in nSolver 3.0. Subsequent analyses were performed using the nCounter Advanced Analysis 2.0 plug-in (NanoString Technologies, Inc. 530 Fairview Ave N, Seattle, Washington, USA). The gene expression normalization was performed using the geNorm algorithm that selected the best housekeeping genes from the initial list of 40 genes (attached). To visualize the results, unsupervised clustering was used to generate heatmap based on the QC passed, normalized data counts of individual genes. Differential expression was graphed as a volcano plot with individual genes −log_10_ (*p*-value) and log_2_ fold change compared to the control group. Pathview module was used to display overexpressed genes (gold color) or downregulated genes (blue color) overlaid on KEGG pathways.

## 3. Results

### 3.1. Global Gene Expression Profile of Trigger Finger Samples Compared to Control

**Figure 1:**
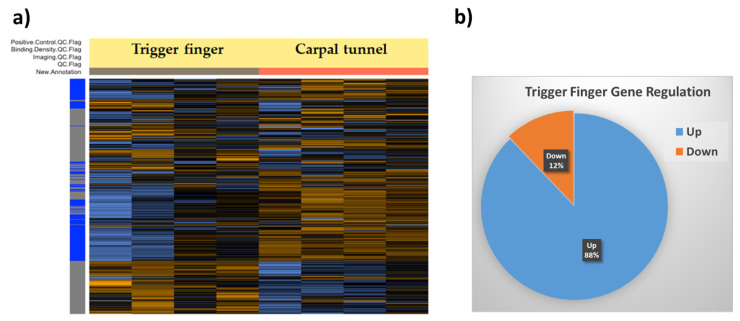
(**a**) Heatmap of normalized data generated via unsupervised clustering by the Nanostring nSolver software. Heatmap is scaled to give all genes equal variance. Control samples (*n* = 4) are organized under the orange column, and trigger finger samples (*n* = 4) are organized under the gray column. Within the gene clusters, orange indicates high expression, and blue indicates low expression. (**b**) Pie chart showing the percentage of genes up- and downregulated in trigger finger compared to carpal tunnel samples.

**Figure 2:**
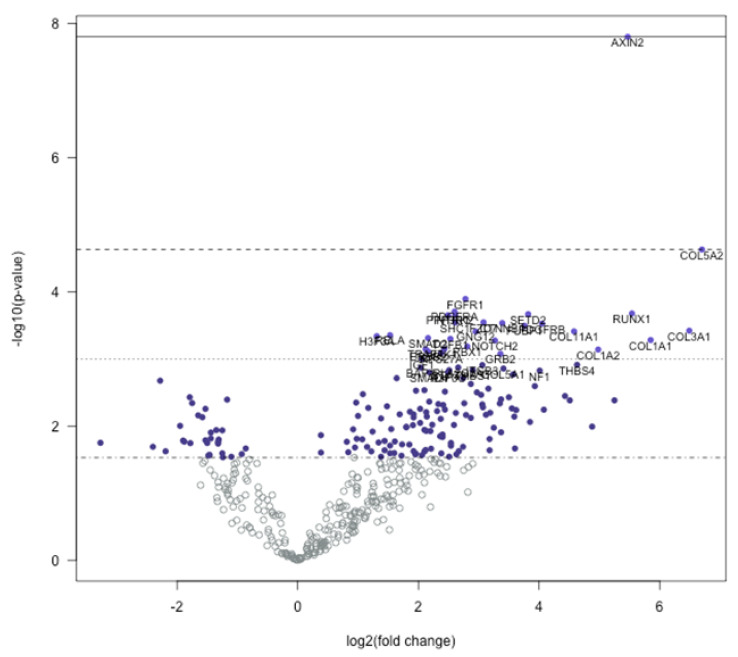
Volcano plot displaying each gene tested plotted comparing −log_10_ (*p*-value) and log_2_ fold change. Horizontal lines on the plot describe statistical significance; thus, highly significant values are at the top of the plot. Highly differentially expressed genes are at the horizontal extremes of the plot. The 40 most statistically significant values are highlighted in the plot.

We used the Nanostring nSolver software to elucidate the differentially regulated genes in trigger finger samples compared to carpal tunnel control samples. The heatmap generated after raw data analysis (Figure 1) indicates distinct expression profiles for both up- and downregulated genes. The volcano plot (Figure 2) shows all samples plotted as a function of fold change vs. *p*-value.

Genes that exhibited a significant (*p* < 0.05) and 1.4-fold change in expression compared to the control group were selected. Overall, 165 genes were differentially regulated; 145 genes were upregulated, whereas 20 were downregulated. The overall fold changes of each of these genes and the pathways they impact are shown in Table 2. It is encouraging that our findings coincide with those previously reported by us [10] and others [12].

The genes with the highest positive fold-change differences were three collagen genes, COL5A2 (6.7), COL3A1 (6.49), and COL1A1 (5.85). In addition to these three upregulated collagen genes, four other collagen transcribing genes were upregulated within the 145 isolated upregulated genes, COL1A2 (4.98), COL11A1 (4.58), COL5A1 (3.41), and COL2A1 (2.67). All upregulated collagen-transcribing genes impacted the PI3K genetic signaling pathway. In addition to these collagen transcribing genes, RUNX1 and IGF1 genes were also upregulated, impacting the common transcriptional misregulation pathway. Other notable upregulated genes included AXIN2 (5.47), PPP3CB (2.76), PPP3R1 (2.49), CCND1 (2.33), SMAD4 (2.18), SMAD2 (2.16), and RAC1 (2.03). These genes all impacted the Wnt signaling pathway.

The gene with the most negative fold-change difference was MMP-3 (−3.27) with a primary impact on the transcriptional misregulation pathway. There were no collagen-transcribing genes with negative fold-change values <−1.40. Other notable genes that were downregulated included NODAL (−2.4) and LEFTY1 (0.00994), both with a primary impact on the TGF-beta signaling pathway.

### 3.2. Signaling Pathway Predictions

The Nanostring nSolver software allowed for signaling pathway prediction through its directed global significance score ratings (Table 3). This statistic measures the extent of up- and downregulation compared to the control of a distinct signaling pathway. In addition to the global significance score ratings, a comprehensive roadmap generated by the Nanostring nSolver software of the genetic pathway known as PathView with both positive and negative regulatory effects is shown in Supplementary Figure S1.

**Figure 3:**
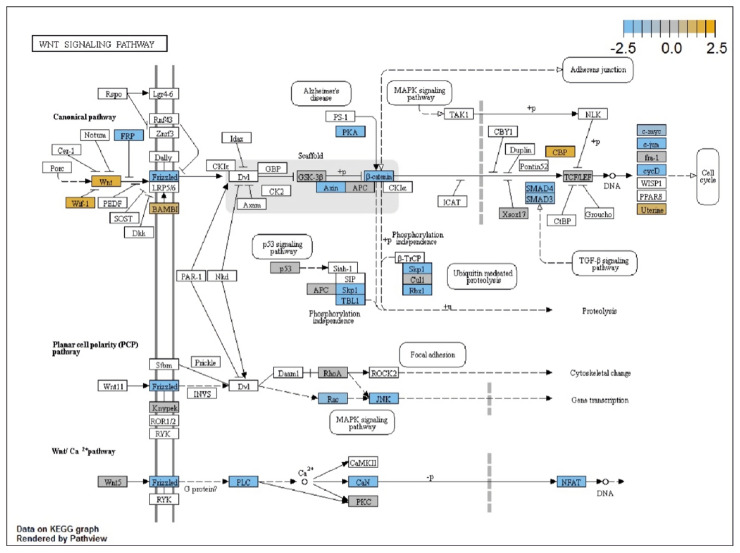
Pathview analysis done by NanoString nSolver software showing a comprehensive pathway roadmap for differentially expressed genes within WNT signaling pathways. Elements overexpressed are shown in gold, elements underexpressed are shown in blue, and elements with unchanged expression are shown in gray.

The pathway with the highest global significance rating in trigger finger samples compared to controls was the Wnt signaling pathway with a score of 6.268 (Figure 3). Other significant upregulated pathways included the PI3K signaling pathway (3.283), the TGF-beta signaling pathway (2.951), and the transcriptional misregulation pathway (2.648). Two pathways with global significance score ratings less than 1 were the chromatin modification pathway (0.579) and the Hedgehog pathway (0.273).

## 4. Discussion

Trigger finger is widely understood as a “mild” hand pathology but is a condition that renders significant pain in patients, which greatly impacts quality of life [23]. The molecular mechanism of the trigger finger and the potential pathways that lead to trigger finger pathogenesis are still unknown. Previously, our group [10] and others [12] demonstrated alteration in extracellular matrix (ECM) (collagen 1a1, collagen 3a1, matrix metallopeptidase (MMP)-2, MMP-3, ADAMTS-5, TIMP-3, aggrecan, biglycan, decorin, and versican) and growth factor (TGF-b and IGF) genes.

Our study utilized the Nanostring nCounter Gene Expression Assay, which simultaneously detects up to 800 genes in a single reaction. We identified 165 statistically significant genes that were differentially regulated in trigger finger, compared to carpal tunnel. To our knowledge, our study is the first study to conduct a comprehensive gene expression analysis on trigger finger to understand its pathogenesis. ECM genes (seven collagens) were significantly upregulated, which is no surprise. Collagens have long been known to be the most abundant fibrous protein in the ECM that provides structural support and cellular strength, along with tissue repair and remodeling capabilities [24,25,26,27]. In the context of tendinopathies, it has been previously reported that collagen types I, III, and V are increased in proportion to other collagens and contribute to the mechanical weakness of the diseased tendon [28,29,30]. Basal production and degradation of collagen is a balanced equilibrium that ensures proper systemic functioning of the ECM and body. This equilibrium is further maintained through the function of MMP enzymes that work to degrade various ECM proteins such as collagens, proteoglycans, and many other ECM components [31,32,33]. In our study, MMP-3 was significantly (−3.27) downregulated in trigger finger samples. MMP-3 is an enzyme that degrades fibronectin, gelatin, and type 1 collagen, among many other ECM components, and it directly activates pro-collagenases such as MMP-1, MMP-7, MMP-8, MMP-9, and MMP-13 [34,35,36]. Thus, the downregulation of MMP-3 has wide-ranging effects that could potentially explain the vast build-up of collagen proteins in trigger finger [37]. Previously, Riley et al. [38] reported that the activity of MMP-3 (compared to MMP-1 and MMP-2 in tendon pathologies) was significantly reduced, which leads to increased turnover and deterioration in the quality of the collagen network [38]. The change in ECM remodeling activity has been known to be associated with an onset of tendinopathy, and this phenomenon could be due to the imbalance between collagen production and MMP-mediated collagen degradation [39]. Thus, the overabundance of collagen can be attributed to decreased MMPs expression, potentially leading to the fibroproliferation of formerly healthy finger tendons and, ultimately, trigger finger.

Fibrosis is defined as the overgrowth, hardening, and/or scarring of tissues due to the abnormal deposition of ECM components, such as collagen [40]. Fibrotic tissue generation is dependent on the production of collagen from myofibroblast cells that are dependent on various signaling pathways triggered by a multitude of genetic factors [5,40,41,42]. In the trigger finger, persistent tissue injury on the pathological flexor tendon eventually triggers fibrosis, but the exact signaling and/or molecular pathway is still a mystery [43,44]. One factor that was considerably upregulated in our study is TGF-β1 (2.53). TGF-β1 is a known stimulator in the molecular pathogenesis of fibrosis in another notable musculoskeletal fibroproliferative hand pathology, Dupuytren’s contracture [42,45,46,47]. In Dupuytren’s contracture, TGF-β1 acts as a growth factor that induces fibroblast contraction within pathological tissues, leading to deformation at the cellular level [48]. Overstimulation of TGF-β1 stimulates the Wnt/β-catenin pathway by decreasing the expression of the Wnt pathway antagonist, Dickkopf-1 [49,50]. Multiple Wnt signaling genes such as RAC1 (2.03), SMAD2 (2.16), SMAD4 (2.18), CCND1 (2.33), PPP3R1 (2.49), PPP3CB (2.76), and AXIN2 (5.47) were significantly upregulated in the trigger finger. Bioinformatics analysis showed that Wnt signaling was the most upregulated cellular pathway, with a directed differential expression rating of 6.268 compared to control. TGF-β1-mediated Wnt signaling has been proven in other studies to regulate fibroproliferation in lung fibrosis, renal fibrosis, skin fibrosis, musculoskeletal fibrosis, and liver fibrosis which could potentially mediate fibrosis in trigger finger [51,52]. Lederhose disease [53,54], adhesive capsulitis [55,56,57], and Peyronie’s disease [45,58,59] are prominent fibroproliferative disorders that share molecular characteristics with Dupuytren’s contracture. We believe that the trigger finger also shares many of the same molecular characteristics as these fibrotic disorders.

We also noted that the “transcriptional misregulation” pathway was upregulated. One of the genes of this pathway, RUNX1 (RUNX family transcription factor 1), was upregulated with a foldchange of 5.54. RUNX1 interacts with other proteins to play important and dynamic roles in ribosome biogenesis, cell-cycle regulation, and TGF-β1 signaling regulation [60,61]. Upregulation of RUNX1 is known to play a role in the increased cellular commitment of mesenchymal stem cells to myofibroblasts [62]. Elevated levels of RUNX1 could lead to many manifestations of the trigger finger: increased myofibroblast activity, increased collagen production, and fibrosis of the finger tendon. Another gene that was upregulated was IGF-1 (insulin-like growth factor 1) (2.06), a known hormone that has diverse roles in regulating growth on almost every cell in the body [63]. In the context of tissue repair, IGF-1 can modulate the conversion of fibroblasts to myofibroblasts and, thus, stimulate the production of collagen [64,65,66,67]. The upregulation of IGF-1 and its downstream effects on collagen production could also contribute to collagen’s overabundance leading to fibrotic tissue generation. Both of these genetic factors, IGF-1 and RUNX1, being a regulatory hormone and a transcription factor, respectively, have a multitude of effects outside of tissue repair and collagen production. Our study identified several genes (Table 1) and signaling pathways (Table 2) dysregulated in the trigger finger and might be involved in the pathogenesis.

Our study had certain limitations. We used a limited number of samples but enough for a proof-of-concept study. Our control group was also not an “actual” control as carpal tunnel tissue is not healthy but diseased tissue. It was complicated to obtain healthy controls due to age-matching restrictions and the ethical limitations of conducting surgery on healthy individuals. Overall, our pilot study found several novel genes and signaling pathways involved in the pathophysiology of trigger finger. The outcome of our study will further help us in understanding the molecular signaling pathways involved in the pathogenesis and designing therapeutic strategies for the treatment of the trigger finger.

## Figures and Tables

**Table 1:** Characteristics of Patients used for tissue samples.

	Patient Group	Patient Age	Patient Gender
Control	Carpal tunnel	35	Female
	Carpal tunnel	37	Female
	Carpal tunnel	44	Female
	Carpal tunnel	51	Female
Experimental	Trigger finger	25	Female
	Trigger finger	46	Female
	Trigger finger	52	Female
	Trigger finger	40	Female

**Table 2:** Table of log_2_ fold change, *p*-value, and genetic pathway impact for 165 genes with fold change values ±1.40. Twenty genes were downregulated, whereas 145 genes were upregulated for trigger finger samples, as compared to controls.

Gene	Fold Change (Log_2_)	*p*-Value	Genetic Pathway Impacted
MMP3-mRNA	−3.27	0.0178	Transcriptional misregulation
NODAL-mRNA	−2.4	0.0204	TGF-beta
HMGA2-mRNA	−2.28	0.00211	Transcriptional misregulation
CACNA1E-mRNA	−2.19	0.0238	MAPK
LEFTY1-mRNA	−1.95	0.00994	TGF-beta
FGF22-mRNA	−1.9	0.0165	MAPK, PI3K, Ras
CASP10-mRNA	−1.88	0.0171	Cell cycle/apoptosis
FGF21-mRNA	−1.79	0.00372	MAPK, PI3K, Ras
KIT-mRNA	−1.78	0.018	Driver gene, PI3K, Ras
FGFR2-mRNA	−1.75	0.00455	Driver gene, MAPK, PI3K, Ras
IL7R-mRNA	−1.65	0.00693	JAK/STAT, PI3K
DKK4-mRNA	−1.58	0.0074	Wnt
WNT2-mRNA	−1.57	0.0359	Hedgehog, Wnt
EFNA3-mRNA	−1.54	0.033	PI3K, Ras
WIF1-mRNA	−1.53	0.00555	Wnt
WNT6-mRNA	−1.53	0.0163	Hedgehog, Wnt
C19orf40-mRNA	−1.5	0.0179	DNA damage repair
HMGA1-mRNA	−1.48	0.0274	Chromatin modification
CREBBP-mRNA	−1.46	0.0269	Cell cycle/apoptosis, chromatin modification, driver gene, JAK/STAT, Notch, TGF-beta, Wnt
CDKN2D-mRNA	−1.45	0.0453	Cell cycle/apoptosis
NF2-mRNA	1.48	0.00508	Driver gene
RELA-mRNA	1.53	0.000445	Cell cycle/apoptosis, MAPK, PI3K, Ras, transcriptional misregulation
PRKDC-mRNA	1.53	0.0196	Cell cycle/apoptosis, DNA damage repair
IL8-mRNA	1.53	0.0255	Transcriptional misregulation
MAD2L2-mRNA	1.53	0.034	Cell cycle/apoptosis, DNA damage repair
GADD45A-mRNA	1.55	0.0111	Cell cycle/apoptosis, MAPK
CIC-mRNA	1.56	0.0437	Driver gene
ITGA9-mRNA	1.58	0.0335	PI3K
SOX9-mRNA	1.6	0.0253	Driver gene
LIFR-mRNA	1.61	0.017	JAK/STAT
RAD21-mRNA	1.64	0.00194	Cell cycle/apoptosis
KRAS-mRNA	1.68	0.0362	Driver gene, MAPK, PI3K, Ras
ITGA2-mRNA	1.68	0.0454	PI3K
MLF1-mRNA	1.69	0.00669	Transcriptional misregulation
CASP3-mRNA	1.71	0.0358	Cell cycle/apoptosis, MAPK
ITGB4-mRNA	1.72	0.0277	PI3K
IL1R1-mRNA	1.73	0.0188	Cell cycle/apoptosis, MAPK
IRAK3-mRNA	1.8	0.0103	Cell cycle/apoptosis
CBL-mRNA	1.8	0.0104	Driver gene, JAK-STAT
PPP2R1A-mRNA	1.85	0.0189	Driver gene, PI3K, TGF-beta
IGFBP3-mRNA	1.86	0.00613	Transcriptional misregulation
JAK2-mRNA	1.88	0.0343	Driver gene, JAK/STAT, PI3K
FLT1-mRNA	1.91	0.00971	PI3K, Ras, transcriptional misregulation
HIST1H3H-mRNA	1.92	0.00669	transcriptional misregulation
NBN-mRNA	1.92	0.00744	DNA damage repair
TGFBR2-mRNA	1.92	0.0237	MAPK, TGF-beta, transcriptional misregulation
PLCB1-mRNA	1.95	0.0264	Wnt
MSH6-mRNA	1.95	0.0378	Driver gene
PPP3CA-mRNA	1.95	0.0439	Cell cycle/apoptosis, MAPK, Wnt
SF3B1-mRNA	1.96	0.00297	Driver gene
PIM1-mRNA	1.96	0.0259	JAK/STAT
SMAD3-mRNA	1.99	0.0401	Cell cycle/apoptosis, TGF-beta, Wnt
RAC1-mRNA	2.03	0.00555	MAPK, PI3K, Ras, Wnt
TNFRSF10B-mRNA	2.03	0.00899	Cell cycle/apoptosis
BAP1-mRNA	2.04	0.00136	Driver gene
PHF6-mRNA	2.05	0.0458	Driver gene
IGF1-mRNA	2.06	0.00101	PI3K, Ras, transcriptional misregulation
CDKN1C-mRNA	2.06	0.0272	Cell cycle/apoptosis
AKT3-mRNA	2.1	0.00292	Cell cycle/apoptosis, JAK/STAT, MAPK, PI3K, Ras
ITGA6-mRNA	2.1	0.0114	PI3K
CHUK-mRNA	2.1	0.024	Cell cycle/apoptosis, MAPK, PI3K, Ras
TRAF7-mRNA	2.12	0.000721	Driver gene
ID2-mRNA	2.12	0.0228	TGF-beta, transcriptional misregulation
PLCB4-mRNA	2.13	0.00622	Wnt
HSPB1-mRNA	2.13	0.0118	MAPK
PLAU-mRNA	2.14	0.00723	Transcriptional misregulation
SMAD2-mRNA	2.16	0.000491	Cell cycle/apoptosis, driver gene, TGF-beta
ERBB2-mRNA	2.16	0.000777	Driver gene
SMAD4-mRNA	2.18	0.0016	Cell cycle/apoptosis, driver gene, TGF-beta, Wnt
SOS2-mRNA	2.18	0.00433	JAK/STAT, MAPK, PI3K, Ras
SMC1A-mRNA	2.19	0.0477	Cell cycle/apoptosis
NFE2L2-mRNA	2.2	0.0119	Driver gene
MAPK3-mRNA	2.21	0.0218	MAPK, PI3K, Ras, TGF-beta
MDM2-mRNA	2.21	0.0312	Driver gene, cell cycle
VHL-mRNA	2.23	0.00957	Driver gene
NUPR1-mRNA	2.26	0.035	Transcriptional misregulation
ATR-mRNA	2.28	0.0314	Cell cycle/apoptosis
DDB2-mRNA	2.31	0.006	DNA damage repair
BMP4-mRNA	2.32	0.0498	Hedgehog, TGF-beta
CCND1-mRNA	2.33	0.00471	Cell cycle/apoptosis, JAK/STAT, PI3K, Wnt
SETBP1-mRNA	2.34	0.0355	Driver gene
SOCS3-mRNA	2.36	0.0142	JAK/STAT
PIK3R1-mRNA	2.37	0.00782	Cell cycle/apoptosis, driver gene, JAK/STAT, PI3K, Ras
KDM5C-mRNA	2.37	0.0363	Driver gene
RPS27A-mRNA	2.38	0.000817	DNA damage repair
MGMT-mRNA	2.38	0.0256	DNA damage repair
GADD45B-mRNA	2.4	0.0134	Cell cycle/apoptosis, MAPK
MAP3K12-mRNA	2.4	0.0146	Chromatin modification, MAPK
PIK3CA-mRNA	2.41	0.00485	Cell cycle/apoptosis, driver gene, JAK/STAT, PI3K, Ras
JAK1-mRNA	2.43	0.000718	Driver gene, JAK/STAT, PI3K
CASP7-mRNA	2.44	0.00307	Cell cycle/apoptosis
UBB-mRNA	2.44	0.00569	DNA damage repair
ITGB8-mRNA	2.47	0.0405	PI3K
PPP3R1-mRNA	2.49	0.000224	Cell cycle/apoptosis, MAPK, Wnt
H3F3C-mRNA	2.49	0.00159	Transcriptional misregulation
STAT3-mRNA	2.51	0.00148	JAK/STAT
BAX-mRNA	2.51	0.0286	Cell cycle/apoptosis
TGFB1-mRNA	2.53	0.000504	Cell cycle/apoptosis, MAPK, TGF-beta
B2M-mRNA	2.54	0.0179	Driver gene
TLR4-mRNA	2.54	0.0197	PI3K
RAF1-mRNA	2.59	0.00964	MAPK, PI3K, Ras
PDGFRA-mRNA	2.6	0.000198	Driver gene, MAPK, PI3K, Ras
NTRK2-mRNA	2.61	0.000221	MAPK
SHC1-mRNA	2.61	0.000287	Ras
IDH2-mRNA	2.62	0.00638	Driver gene
ID1-mRNA	2.63	0.0264	TGF-beta
PLA2G2A-mRNA	2.66	0.00135	Ras
COL2A1-mRNA	2.67	0.0237	PI3K
WHSC1-mRNA	2.74	0.00194	Transcriptional misregulation
AKT1-mRNA	2.74	0.0204	Cell cycle/apoptosis, driver gene, JAK/STAT, MAPK, PI3K, Ras
MMP9-mRNA	2.75	0.0457	Transcriptional misregulation
PPP3CB-mRNA	2.76	0.00458	Cell cycle/apoptosis, MAPK, Wnt
FGFR1-mRNA	2.78	0.000128	MAPK, PI3K, Ras
MAP2K2-mRNA	2.79	0.00685	MAPK, PI3K, Ras
RBX1-mRNA	2.81	0.000656	Cell cycle/apoptosis, TGF-beta, Wnt
JUN-mRNA	2.83	0.0409	MAPK, Wnt
SKP1-mRNA	2.87	0.00236	Cell cycle/apoptosis, TGF-beta, Wnt
ABL1-mRNA	2.87	0.00756	Cell cycle/apoptosis, driver gene, Ras
THBS1-mRNA	2.9	0.00147	PI3K, TGF-beta
KLF4-mRNA	2.9	0.0365	Driver gene
GNG12-mRNA	2.95	0.000392	MAPK, PI3K, Ras
PDGFD-mRNA	2.97	0.00315	PI3K, Ras
CHAD-mRNA	3.04	0.00343	PI3K
ITGB3-mRNA	3.06	0.00124	PI3K
BCL2L1-mRNA	3.06	0.00476	Cell cycle/apoptosis, JAK/STAT, PI3K, Ras, transcriptional misregulation
NCOR1-mRNA	3.07	0.00525	Driver gene, transcriptional misregulation
FZD7-mRNA	3.08	0.000286	Wnt
POLD4-mRNA	3.12	0.0068	DNA damage repair
PIK3R2-mRNA	3.16	0.00277	Cell cycle/apoptosis, JAK/STAT, PI3K, Ras
TGFB3-mRNA	3.18	0.0156	Cell cycle/apoptosis, MAPK, TGF-beta
PRKACA-mRNA	3.18	0.023	Cell cycle/apoptosis, Hedgehog, MAPK, Ras, Wnt
TBL1XR1-mRNA	3.19	0.00635	Wnt
GNAS-mRNA	3.25	0.0106	Driver gene
NOTCH2-mRNA	3.27	0.000534	Driver gene, Notch
COMP-mRNA	3.35	0.00461	PI3K
GRB2-mRNA	3.36	0.000844	JAK/STAT, MAPK, PI3K, Ras
CREB3L1-mRNA	3.37	0.004	PI3K
CAPN2-mRNA	3.37	0.0124	Cell cycle/apoptosis
CTNNB1-mRNA	3.39	0.000293	Driver gene, Wnt
COL5A1-mRNA	3.41	0.0014	PI3K
MAPK1-mRNA	3.5	0.00372	MAPK, PI3K, Ras, TGF-beta
GAS1-mRNA	3.55	0.00546	Hedgehog
ASXL1-mRNA	3.57	0.00171	Driver gene
HSP90B1-mRNA	3.59	0.00724	PI3K
FLNA-mRNA	3.6	0.0216	MAPK
FGF18-mRNA	3.62	0.00578	MAPK, PI3K, Ras
FUBP1-mRNA	3.76	0.000322	Driver gene
SETD2-mRNA	3.82	0.000216	Driver gene
FOS-mRNA	3.85	0.0087	MAPK
NFATC1-mRNA	3.93	0.00254	MAPK, Wnt
NF1-mRNA	4.01	0.0015	Driver gene, MAPK, Ras
PDGFRB-mRNA	4.05	0.000298	MAPK, PI3K, Ras
LTBP1-mRNA	4.08	0.00571	TGF-beta
NFKBIZ-mRNA	4.43	0.00357	Transcriptional misregulation
SFRP2-mRNA	4.51	0.00414	Wnt
COL11A1-mRNA	4.58	0.00039	PI3K
THBS4-mRNA	4.63	0.00123	PI3K
FN1-mRNA	4.88	0.0102	PI3K
COL1A2-mRNA	4.98	0.000728	PI3K
SFRP4-mRNA	5.25	0.00414	Wnt
AXIN2-mRNA	5.47	1.58 × 10^−8^	Wnt
RUNX1-mRNA	5.54	0.000211	Driver gene, transcriptional misregulation
COL1A1-mRNA	5.85	0.000525	PI3K
COL3A1-mRNA	6.49	0.000381	PI3K
COL5A2-mRNA	6.7	2.35 × 10^−5^	PI3K

**Table 3:** Global significance ratings comparing overall differential expression of selected pathways relative to control.

Differential Expression in Trigger Finger vs. Baseline of Carpal Tunnel	
**Wnt**	6.268
**Driver Gene**	3.382
**PI3K**	3.283
**MAPK**	3.086
**Ras**	3.053
**TGF-Beta**	2.951
**Cell Cycle—Apoptosis**	2.719
**Transcriptional Misregulation**	2.648
**JAK-STAT**	2.479
**Notch**	1.94
**DNA Damage—Repair**	1.625
**hromatin Modification**	0.579
**Hedgehog**	0.273

## Data Availability

The data that support the findings of this study will be available from the corresponding author, upon reasonable request.

## Supplementary Materials

The following are available online at https://www.mdpi.com/article/10.3390/healthcare9111592/s1, Table S1: Gene and Probe Information. Figure S1: Pathview analysis done by NanoString nSolver software showing a comprehensive pathway roadmap for differentially expressed genes within various KEGG pathways of our samples. Elements over-expressed are shown in gold, elements under-expressed are shown in blue, and elements with unchanged expression are shown in gray.
