# Supplementary material for: Nanostring-Based Identification of the Gene Expression Profile in Trigger Finger Samples
Source: Healthcare (Basel). 2021 Nov 20;9(11):1592. doi: 10.3390/healthcare9111592 (PMC8619339; doi:10.3390/healthcare9111592)
Supplement: Supplementary file 1 [file healthcare-09-01592-s001.zip › healthcare-1359505 figure S1 final.pdf]

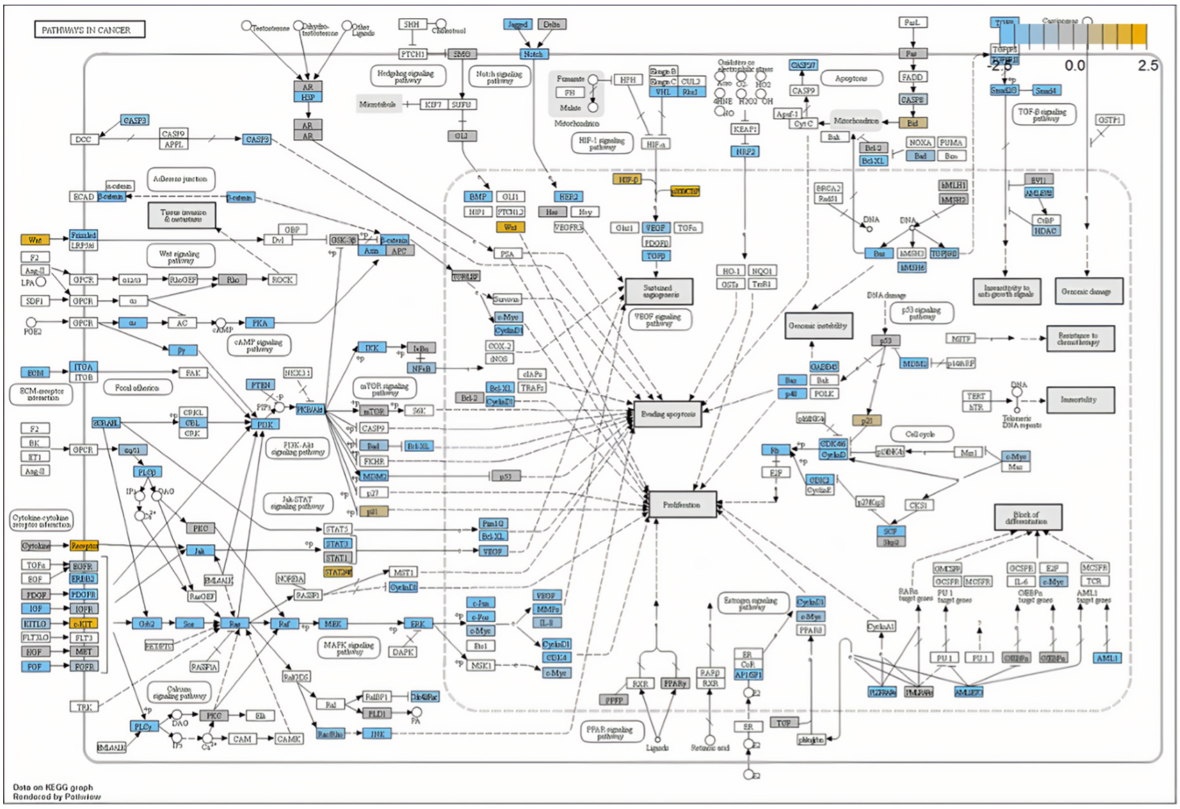

Figure S1. Pathview analysis done by NanoString nSolver software showing a comprehensive pathway roadmap for differentially expressed genes within various KEGG pathways of our samples. Elements over-expressed are shown in gold, elements under-expressed are shown in blue, and elements with unchanged expression are shown in gray.
